# Multi-Reader Multi-Case Studies Using the Area under the Receiver Operator Characteristic Curve as a Measure of Diagnostic Accuracy: Systematic Review with a Focus on Quality of Data Reporting

**DOI:** 10.1371/journal.pone.0116018

**Published:** 2014-12-26

**Authors:** Thaworn Dendumrongsup, Andrew A. Plumb, Steve Halligan, Thomas R. Fanshawe, Douglas G. Altman, Susan Mallett

**Affiliations:** 1 Department of Radiology, Prince of Songkla University, Hat Yai, Thailand; 2 Centre for Medical Imaging, University College London, London, United Kingdom; 3 Nuffield Department of Primary Care Health Sciences, Oxford University, Oxford, United Kingdom; 4 Centre for Statistics in Medicine, Wolfson College, Oxford University, Oxford, United Kingdom; University of Geneva, Switzerland

## Abstract

**Introduction:**

We examined the design, analysis and reporting in multi-reader multi-case (MRMC) research studies using the area under the receiver-operating curve (ROC AUC) as a measure of diagnostic performance.

**Methods:**

We performed a systematic literature review from 2005 to 2013 inclusive to identify a minimum 50 studies. Articles of diagnostic test accuracy in humans were identified via their citation of key methodological articles dealing with MRMC ROC AUC. Two researchers in consensus then extracted information from primary articles relating to study characteristics and design, methods for reporting study outcomes, model fitting, model assumptions, presentation of results, and interpretation of findings. Results were summarized and presented with a descriptive analysis.

**Results:**

Sixty-four full papers were retrieved from 475 identified citations and ultimately 49 articles describing 51 studies were reviewed and extracted. Radiological imaging was the index test in all. Most studies focused on lesion detection vs. characterization and used less than 10 readers. Only 6 (12%) studies trained readers in advance to use the confidence scale used to build the ROC curve. Overall, description of confidence scores, the ROC curve and its analysis was often incomplete. For example, 21 (41%) studies presented no ROC curve and only 3 (6%) described the distribution of confidence scores. Of 30 studies presenting curves, only 4 (13%) presented the data points underlying the curve, thereby allowing assessment of extrapolation. The mean change in AUC was 0.05 (−0.05 to 0.28). Non-significant change in AUC was attributed to underpowering rather than the diagnostic test failing to improve diagnostic accuracy.

**Conclusions:**

Data reporting in MRMC studies using ROC AUC as an outcome measure is frequently incomplete, hampering understanding of methods and the reliability of results and study conclusions. Authors using this analysis should be encouraged to provide a full description of their methods and results.

## Introduction

The receiver operator characteristic (ROC) curve describes a plot of sensitivity versus 1-specificity for a diagnostic test, across the whole range of possible diagnostic thresholds [1]. The area under the ROC curve (ROC AUC) is a well-recognised single measure that is often used to combine elements of both sensitivity and specificity, sometimes replacing these two measures. ROC AUC is often used to describe the diagnostic performance of radiological tests, either to compare the performance of different tests or the same test under different circumstances [2], [3]. Radiological tests must be interpreted by human observers and a common study design uses multiple readers to interpret multiple image cases; the multi-reader multi-case (MRMC) design [4]. The MRMC design is popular because once a radiologist has viewed 20 cases there is less information to be gained by asking him to view a further 20 than by asking a different radiologist to view the same 20. This procedure enhances the generalisability of study results and having multiple readers interpret multiple cases enhances statistical power. Because multiple radiologists view the same cases, “clustering” occurs. For example, small lesions are generally seen less frequently than larger lesions, i.e. reader observations are clustered within cases. Similarly, more experienced readers are likely to perform better across a series of cases than less experienced readers, i.e. results are correlated within readers. Bootstrap resampling and multilevel modeling can account for clustering, linking results from the same observers and cases, so that 95% confidence intervals are not too narrow. MRMC studies using ROC AUC as the primary outcome are often required by regulatory bodies for the licensing of new radiological devices [5].

We attempted to use ROC AUC as the primary outcome measure in a prior MRMC study of computer-assisted detection (CAD) for CT colonography [6]. However, we encountered several difficulties when trying to implement this approach, described in detail elsewhere [7]. Many of these difficulties were related to issues implementing confidence scores in a transparent and reliable fashion, which led ultimately to a flawed analysis. We considered, therefore, that for ROC AUC to be a valid measure there are methodological components that need addressing in study design, data collection and analysis, and interpretation. Based on our attempts to implement the MRMC ROC AUC analysis, we were interested in whether other researchers have encountered similar hurdles and, if so, how these issues were tackled.

In order to investigate how often other studies have addressed and reported on methodological issues with implementing ROC AUC, we performed a systematic review of MRMC studies using ROC AUC an outcome measure. We searched and investigated the available literature with the objective to describe the statistical methods used, the completeness of data presentation, and investigate whether any problems with analysis were encountered and reported.

## Methods

### Ethics statement

Ethical approval is not required by our institutions for research studies of published data.

### Search strategy, inclusion and exclusion criteria

This systematic review was performed guided by the Preferred Reporting Items for Systematic Reviews and Meta-Analyses (PRISMA), an evidence-based minimum set of items for reporting in systematic reviews and meta-analyses [8]. We developed an extraction sheet for the systematic review, broken down into different sections (used as subheadings for the Results section of this report), with notes relating to each individual item extracted (S1 File). In consensus we considered approximately 50 articles would provide a sufficiently representative overview of current reporting practice. Based on our prior experience of performing systematic reviews we believed that searching for additional articles beyond 50 would be unlikely to yield valuable additional data (i.e. we believed we would reach “saturation” by 50 articles) yet would present a very considerable extraction burden.

In order to achieve this, potentially eligible primary articles published between 2005 and February 2013 inclusive were identified by a radiologist researcher (TD) using PUBMED via their citation of one or more of 8 key methodological articles relating to MRMC ROC AUC analysis [9]–[16]. To achieve this the Authors' names (combined using “AND”) were entered in the PUBMED search field and the specific article identified and clicked in the results list. The abstract was then accessed and the “Cited By # PubMed Central Articles” link and “Related Citations” link used to identify those articles in the PubMed Central database that have cited the original article. There was no language restriction. Online abstracts were examined in reverse chronological order, the full text of potentially eligible papers then retrieved, and selection stopped once the threshold of 50 studies fulfilling inclusion criteria had been passed.

To be eligible, primary studies had to be diagnostic test accuracy studies of human observers interpreting medical image data from real patients, and attempting to use a MRMC ROC AUC analysis as a study outcome based on the following methodological approaches [9]–[16]; Reviews, solely methodological papers, and those using simulated imaging data were excluded.

### Data extraction

An initial pilot sample of 5 full-paper articles were extracted and the data checked by a subgroup of investigators in consensus, to both confirm the process was feasible and to identify potential problems. These papers were extracted by TD using the search strategy described in the previous section. A further 10 full-papers were extracted by two radiologist researchers again using the same search strategy and working independently (TD, AP) to check agreement further. The remaining articles included in the review were extracted predominantly by TD, who discussed any concerns/uncertainty with AP. Any disagreement following their discussion was arbitrated by SH and/or SM where necessary. These discussions took place during two meetings when the authors met to discuss progress of the review; multiple papers and issues were discussed on each occasion.

The extraction covered the following broad topics: Study characteristics, methods to record study outcomes, model assumptions, model fitting, data presentation (S1 File).

We extracted data relating to the organ and disease studied, the nature of the diagnostic task (e.g. characterization vs. localization vs. presence/absence), test methods, patient source and characteristics, study design (e.g. prospective/retrospective, secondary analysis, single/multicenter) and reference standard. We extracted the number of readers, their prior experience, specific interpretation training for the study (e.g. use of CAD software), blinding to clinical data and/or reference results, the number of times they read each case and the presence of any washout period to diminish recall bias, case ordering, and whether all readers read all cases (i.e. a fully-crossed design). We extracted the unit of analysis (e.g. patient vs. organ vs. segment), and sample size for patients with and without pathology.

We noted whether study imaging reflected normal daily clinical practice or was modified for study purposes (e.g. restricted to limited images). We noted the confidence scores used for the ROC curve and their scale, and whether training was provided for scoring. We noted if there were multiple lesions per unit of analysis. We noted if scoring differed for positive and negative patient cases, whether score distribution was reported, and whether transformation to a normal distribution was performed.

We extracted if ROC cures were presented in the published article and, if so, whether for individual readers, whether the curve was smoothed, and if underlying data points were shown. We defined unreasonable extrapolation as an absence of data in the right-hand 25% of the plot space. We noted the method for curve fitting and whether any problems with fitting were reported, and the method used to compare AUC or pAUC. We extracted the primary outcome, the accuracy measures reported, and whether these were overall or for individual readers. We noted the size of any change in AUC, whether this was significant, and made a subjective assessment of whether significance could be attributed to a single reader or case. We noted how the study authors interpreted change in AUC, if any, and whether any change was reported in terms of effect on individual patients. We also noted if a ROC researcher was named as an author or acknowledged, defined as an individual who had published indexed research papers dealing with ROC methodology.

### Analysis

Data were summarized in an Excel worksheet (Excel For Mac 14.3.9, Microsoft Corporation) with additional cells for explanatory free text. A radiologist researcher (SH) then compiled the data and extracted frequencies, consulting the two radiologists who performed the extraction for clarification when necessary. The investigator group discussed the implication of the data subsequently, to guide interpretation.

## Results

**Figure 1:**
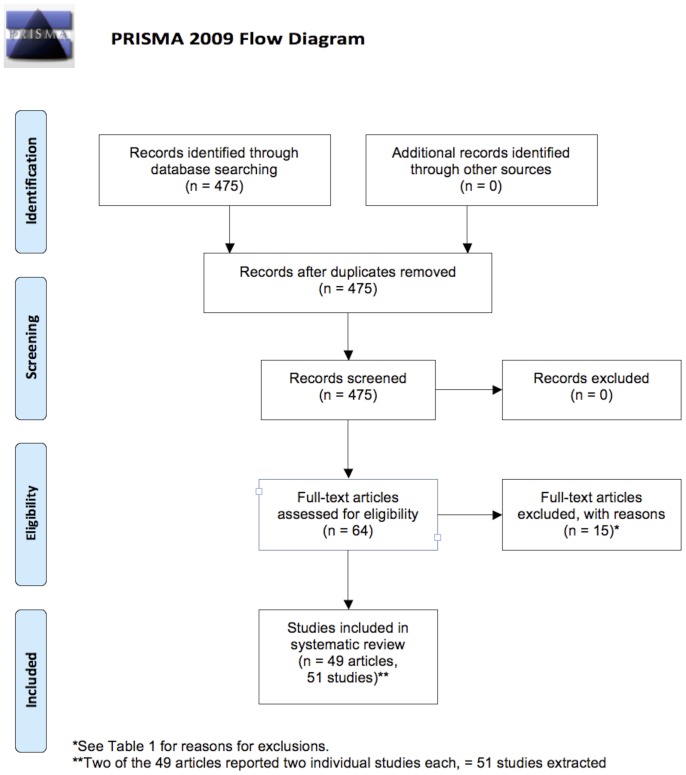
PRISMA flow diagram [8] for the systematic review.

Four hundred and seventy five citations of the 8 key methodological papers were identified and 64 full papers retrieved subsequently. Fifteen [17]–[31] of these were rejected after reading the full text (the papers and reason for rejection are shown in Table 1) leaving 49 [32]–[80] for extraction and analysis that were published between 2010 and 2012 inclusive; these are detailed in Table 1. Two papers [61], [75] contributed two separate studies each, meaning that 51 studies were extracted in total. The PRISMA checklist [8] is detailed in Fig. 1. The raw extracted data are available in S2 File.

**Table 1:** Citations for the 49 papers (contributing 51 studies) included in the systematic review.

Included Articles (first author, year)	
Aoki, 2011 [32]	
Aoki, 2012 [33]	
Berg, 2012 [34]	
Bilello, 2010 [35]	
Choi, 2012 [36]	
Cole, 2012 [37]	
Collettini, 2012 [38]	
Dachman, 2010 [39]	
Dromain, 2012 [40]	
Gennaro, 2010 [41]	
Hupse, 2013 [42]	
Kelly, 2010 [43]	
Kim, 2012 [44]	
Kim, 2010 [45]	
Li, 2012 [46]	
Li, 2011a [47]	
Li, 2011b [48]	
Matsushima, 2010 [49]	
McNulty, 2012 [50]	
Medved, 2011 [51]	
Mermuys, 2010 [52]	
Moin, 2010 [53]	
Muramatsu, 2010 [54]	
Noroozian, 2012 [55]	
Ohgiya, 2012 [56]	
Otani, 2012 [57]	
Padilla, 2013 [58]	
Pollard, 2012 [59]	
Purysko, 2012 [60]	
Rafferty, 2013 [61]	Contributed two studies
Saade, 2013 [62]	
Salazar, 2011 [63]	
Shimauchi, 2011 [64]	
Shiraishi, 2010 [65]	
Subhas, 2011 [66]	
Sung, 2010 [67]	
Svahn, 2012 [68]	
Takahashi, 2010 [69]	
Tan, 2012 [70]	
Timp, 2010 [71]	
Toomey, 2010 [72]	
Uchiyama, 2012 [73]	
Visser, 2012 [74]	
Wallis, 2012 [75]	Contributed two studies
Wardlaw, 2010 [76]	
Way, 2010 [77]	
Yamada, 2011 [78]	
Yamada, 2012 [79]	
Yoshida, 2013 [80]	

Excluded Articles (first author, year)	Primary reason for exclusion
Warren, 2012 [17]	Simulated imaging
Berbaum, 2012 [18]	Simulated imaging
Destounis, 2011 [19]	No MRMC ROC analysis
Jinzaki, 2011 [20]	No MRMC ROC analysis
Krupnski, 2012 [21]	Simulated imaging
Leong, 2012 [22]	Simulated imaging
Nishida, 2011 [23]	No MRMC ROC analysis
Obuchowski, 2010 [24]	Non sequential design
Okamoto, 2011 [25]	No human readers
Reed, 2011 [26]	Simulated imaging
Svane, 2011 [27]	No MRMC ROC analysis
Szucs-Farkas, 2010 [28]	No MRMC ROC analysis
Webb, 2011 [29]	Simulated imaging
Yakabe, 2010 [30]	Simulated imaging
Zanca, 2012 [31]	Not a primary study with original data

Details are also provided for the 15 articles excluded from the systematic review after reading the full-text, along with primary reasons for their exclusion (multiple reasons for exclusion were possible).

### Study characteristics

The index test was imaging in all studies. Breast was the commonest organ studied (20 studies), followed by lung (11 studies) and brain (7 studies). Mammography (15 studies) was the commonest individual modality investigated, followed by plain film (12 studies), CT and MRI (11 studies each), tomosynthesis (six studies), ultrasound (two studies) and PET (one study); 9 studies investigated multiple modalities. In most studies (28 studies) the prime interpretation task was lesion detection. Eleven studies focused on lesion characterization and 12 combined detection and characterization. Forty-one studies compared 2 tests/conditions (i.e. a single test but used in different ways) to a reference standard (41 studies), while 2 studies compared 1 test/condition, 7 studies compared 3 tests/conditions, and 1 study compared 4 tests/conditions. Twenty-five studies combined data to create a reference standard while the reference was a single finding in 24 (14 imaging, 5 histology, 5 other – e.g. endoscopy). The reference method was unclear in 2 studies [54], [55].

Twenty-four studies were single center, 12 multicenter, with the number of centers unclear in 15 (29%) studies. Nine studies recruited symptomatic patients, 8 asymptomatic, and 7 a combination, but the majority (53%; 27 studies) did not state whether patients were symptomatic or not. 42 (82%) studies described the origin of patients with half of these stating a precise geographical region or hospital name. However, 9 (18%) studies did not sufficiently describe the source of patients and 21 (41%) did not describe patients' age and/or gender distribution.

### Study design

**Figure 2:**
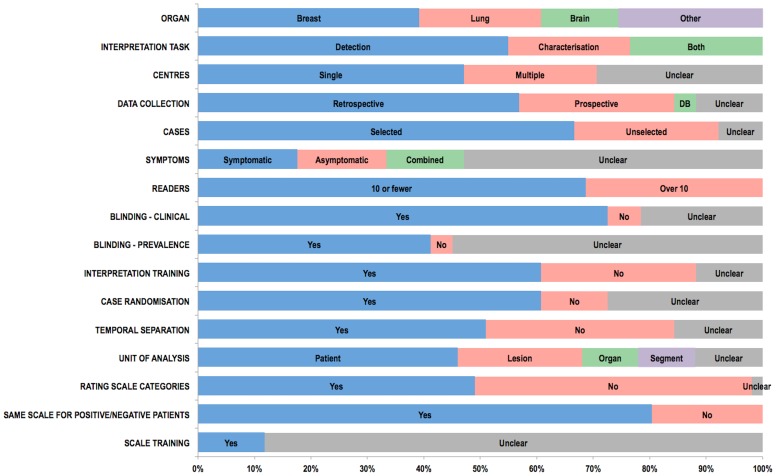
Bar chart showing data extracted by the systematic review relating to study readers, design, and the confidence scales used to build ROC curves.

Extracted data relating to study design and readers are presented graphically in Fig. 2. Most studies (29; 57%) used patient data collected retrospectively. Fourteen (28%) were prospective while 2 used an existing database. Whether prospective/retrospective data was used was unstated/unclear in a further 6 (12%). While 13 studies (26%) used cases unselected other than for the disease in question, the majority (34; 67%) applied further criteria, for example to preselect “difficult” cases (11 studies), or to enrich disease prevalence (4 studies). How this selection bias was applied was stated explicitly in 18 (53%) of these 34. Whether selection bias was used was unclear in 4 studies.

The number of readers per study ranged from 2 [56] to 258 [76]. The mean number was 13, median 6. The large majority of studies (35; 69%) used fewer than 10 readers. Reader experience was described in 40 (78%) studies but not in 11. Specific reader training for image interpretation was described in 31 (61%) studies. Readers were not trained specifically in 14 studies and in 6 it was unclear whether readers were trained specifically or not. Readers were blind to clinical information for individual patients in 37 (73%) studies, unblind in 3, and this information was unrecorded or uncertain in 11 (22%). Readers were blind to prevalence in the dataset in 21 (41%) studies, unblind in 2, but this information was unsure/unrecorded or uncertain in the majority (28, 55%).

Observers read the same patient case on more than one occasion in 50 studies; this information was unclear in the single further study [70]. A fully crossed design (i.e. all readers read all patients with all modalities) was used in 47 (92%) studies, but not stated explicitly in 23 of these. A single study [72] did not use a fully crossed design and the design was unclear or unrecorded in 3 [34], [70], [76]. Case ordering was randomised (either a different random order across all readers or a different random order for each individual reader) between consecutive readings in 31 (61%) studies, unchanged in 6, and unclear/unrecorded in 14 (27%). The ordering of the index test being compared varied between consecutive readings in 20 (39%) studies, was unchanged in 17 (33%), and was unclear/unrecorded in 14 (27%). 26 (51%) studies employed a time interval between readings that ranged from 3 hours [50] to 2 months [63], with a median of 4 weeks. There was no interval (i.e. reading of cases in all conditions occurred at the same sitting) in 17 (33%) studies, and time interval was unclear/unrecorded in 8 (16%).

### Methods of reporting study outcomes

The unit of analysis for the ROC AUC analysis was the patient in 23 (45%) studies, an organ in 5, an organ segment in 5, a lesion in 11 (22%), other in 2, and unclear or unrecorded in 6 (12%); one study [34] examined both organ and lesion so there were 52 extractions for this item. Analysis was based on multiple images in 33 (65%) studies, a single image in 16 (31%), multiple modalities in a single study [40], and unclear in a single study [57]; no study used videos.

The number of disease positive patients per study ranged between 10 [79] and 100 [53] (mean 42, median 48) in 46 studies, and was unclear/unrecorded in 5 studies. The number of disease positive units of outcome for the primary ROC AUC analysis ranged between 10 [79] and 240 [41] (mean 59, median 50) in 43 studies, and was unclear/unrecorded in 8 studies. The number of disease negative patients per study ranged between 3 [69] and 352 [34] (mean 66, median 38) in 44 studies, was zero in 1 study [80], and was unclear/unrecorded in 6 studies. The number of disease negative units of analysis for the primary outcome for the ROC AUC analysis ranged between 10 [51] and 535 [39] (mean 99, median 68) in 42 studies, and was unclear/unrecorded in the remaining 9 studies. The large majority of studies (41, 80%) presented readers with an image or set of images reflecting normal clinical practice whereas 10 presented specific lesions or regions of interest to readers.

Calculation of ROC AUC requires the use of confidence scores, where readers rate their confidence in the presence of a lesion or its characterization. In our previous study [6] we identified the assignment of confidence scores to be potentially on separate scales for disease positive and negative cases [7]. For rating scores used to calculate ROC AUC, 25 (49%) studies used a relatively small number of categories (defined as up to 10) and 25 (49%) used larger scales or a continuous measurement (e.g. visual analogue scale). One study did not specify the scale used [76]. Only 6 (12%) studies stated explicitly that readers were trained in advance to use the scoring system, for example being encouraged to use the full range available. In 15 (29%) studies there was the potential for multiple abnormalities in each unit of analysis (stated explicitly by 12 of these). This situation was dealt with by asking readers to assess the most advanced or largest lesion (e.g. [43]), by an analysis using the highest score attributed (e.g. [42]), or by adopting a per-lesion analysis (e.g. [52]). For 23 studies only a single abnormality per unit of analysis was possible, whereas this issue was unclear in 13 studies.

### Model assumptions

The majority of studies (41, 80%) asked readers to ascribe the same scoring system to both disease-positive and disease-negative patients. Another 9 studies asked that different scoring systems be used, depending on whether the case was perceived as positive or negative (e.g. [61]), or depending on the nature of the lesion perceived (e.g. [66]). Scoring was unclear in a single study [76]. No study stated that two types of true-negative classifications were possible (i.e. where a lesion was seen but misclassified vs. not being seen at all), a situation that potentially applied to 22 (43%) of the 51 studies. Another concern occurs when more than one observation for each patient is included in the analysis, violating the assumption that data are independent. This could occur if multiple diseased segments were analysed for each patient without using a statistical method that treats these as clustered data. An even more flawed approach occurs when analysis includes one segment for patients without disease but multiple segments for patients with disease.

When publically available DBM MRMC software [81] is used for ROC AUC modeling, this requires assumptions of normality for confidence scores or their transformations if the standard parametric ROC curve fitting methods are used. When scores are not normally distributed, even if non parametric approaches are used to estimate ROC AUC, this lack of normality may indicate additional problems with obtaining reliable estimates of ROC AUC [82]–[86]. While 17 studies stated explicitly that the data fulfilled the assumptions necessary for modeling, none described whether confidence scores were transformed to a normal distribution for analysis. Indeed, only 3 studies [54], [73], [76] described the distribution of confidence scores, which was non-normal in each case.

### Model fitting

Thirty (59%) studies presented ROC curves based on confidence scores; i.e. 21 (41%) studies showed no ROC curve. Of the 30 with curves, only 5 presented a curve for each reader whereas 24 presented curves averaged over all readers; a further study presented both. Of the 30 studies presenting ROC curves, 26 (87%) showed only smoothed curves, with the data points underlying the ROC curve presented in only 4 (13%) [43], [51], [63], [78]. Thus, a ROC curve with underlying data points was presented in only 4 of 51 (8%) studies overall. The degree of extrapolation is critical in understanding the reliability of the ROC AUC result [7]. However, extrapolation could only be assessed in these four articles, with unreasonable extrapolation, by our definition, occurring in two [43], [63].

The majority of studies (31, 61%) did not specify the method used for curve fitting. Of the 20 that did, 7 used non-parametric methods (Trapezoidal/Wilcoxon), 8 used parametric methods (7 of which used Proproc), 3 used other methods, and 2 used a combination. Previous research [7], [84] has demonstrated considerable problems fitting ROC curves due to degenerate data where the fitted ROC curve corresponds to vertical and horizontal lines, e.g there are no FP data. Only 2 articles described problems with curve fitting [55], [61]. Two studies stated that data was degenerate: Subhas and co-workers [66] stated that, “data were not well dispersed over the five confidence level scores”. Moin and co-workers [53] stated that, “If we were to recode categories 1 and 2, and discard BI-RADS 0 in the ROC analysis, it would yield degenerative results because the total number of cases collected would not be adequate”. While all studies used MRMC AUC methods to compare AUC outcomes, 5 studies also used other methods (e.g. t-testing) [37], [52], [60], [67], [77]. Only 3 studies described using a partial AUC [42], [55], [77]. Forty-four studies additionally reported non-AUC outcomes (e.g. McNemar's test to compare test performance at a specified diagnostic threshold [58], Wilcoxon signed rank test to compare changes in patient management decisions [64]). Eight (16%) of the studies included a ROC researcher as an author [39], [47], [48], [54], [60], [65], [66], [72].

### Presentation of results

**Figure 3:**
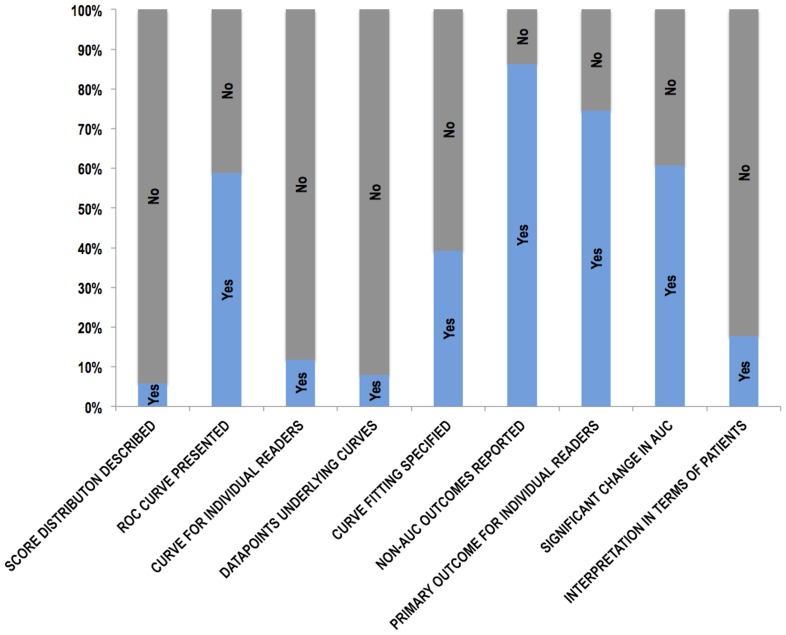
Bar chart showing data extracted by the systematic review relating to the presentation of individual study results.

Extracted data relating to the presentation of individual study results is presented graphically in Fig. 3. All studies presented ROC AUC as an accuracy measure with 49 (96%) presenting the change in AUC for the conditions tested. Thirty-five (69%) studies presented additional measures such as change in sensitivity/specificity (24 studies), positive/negative predictive values (5 studies), or other measures (e.g. changes in clinical management decisions [64], intraobserver agreement [36]). Change in AUC was the primary outcome in 45 (88%) studies. Others used sensitivity [34], [40], accuracy [35], [69], the absolute AUC [44] or JAFROC figure of merit [68]. All studies presented an average of the primary outcome over all readers, with individual reader results presented in 38 (75%) studies but not in 13 (25%). The mean change/difference in AUC was 0.051 (range −0.052 to 0.280) across the extracted studies and was stated as “significant” in 31 and “non-significant” in the remaining 20. No study failed to comment on significance of the stated change/difference in AUC. In 22 studies we considered that a significant change in AUC was unlikely to be due to results from a single reader/patient but we could not determine whether this was possible in 11 studies, and judged this not-applicable in a further 18 studies. One study appeared to report an advantage for a test when the AUC increased, but not significantly [65]. There were 5 (10%) studies where there appeared to be discrepancies between the data presented in the abstract/text/ROC curve [36], [38], [69], [77], [80].

While the majority of studies (42, 82%) did not present an interpretation of their data framed in terms of changes to individual patient diagnoses, 9 (18%) did so, using outcomes in addition to ROC AUC: For example, as a false-positive to true-positive ratio [35] or the proportion of additional biopsies precipitated and disease detected [64], or effect on callback rate [43]. The change in AUC was non-significant in 22 studies and in 12 of these the authors speculated why, for example stating that the number of cases was likely to be inadequate [65], [70], that the observer task was insufficiently taxing [36], or that the difference was too subtle to be resolved [45]. For studies where a non-significant change in AUC was observed, authors sometimes framed this as demonstrating equivalence (16 studies, e.g. [55], [74]), stated that there were other benefits (3 studies), or adopted other interpretations. For example, one study stated that there were “beneficial” effects on many cases despite a non-significant change in AUC [54] and one study stated that the intervention “improved visibility” of microcalcifications noting that the lack of any statistically significant difference warranted further investigation [65].

## Discussion

While many studies have used ROC AUC as an outcome measure, very little research has investigated how these studies are conducted, analysed and presented. We could find only a single existing systematic review that has investigated this question [87]. The authors stated in their Introduction, “we are not aware of any attempt to provide an overview of the kinds of ROC analyses that have been most commonly published in radiologic research.” They investigated articles published in the journal “Radiology” between 1997 and 2006, identifying 295 studies [87]. The authors concluded that “ROC analysis is widely used in radiologic research, confirming its fundamental role in assessing diagnostic performance”. For the present review, we wished to focus on MRMC studies specifically, since these are most complex and are often used as the basis for technology licensing. We also wished to broaden our search criteria beyond a single journal. Our systematic review found that the quality of data reporting in MRMC studies using ROC AUC as an outcome measure was frequently incomplete and who would therefore agree with the conclusions of Shiraishi et al. who stated that studies, “were not always adequate to support clear and clinically relevant conclusions” [87].

Many omissions we identified were those related to general study design and execution, and are well-covered by the STARD initiative [88] as factors that should be reported in studies of diagnostic test accuracy in general. For example, we found that the number of participating research centres was unclear in approximately one-third of studies, that most studies did not describe whether patients were symptomatic or asymptomatic, that criteria applied to case selection were sometimes unclear, and that observer blinding was not mentioned in one-fifth of studies. Regarding statistical methods, STARD states that studies should, “describe methods for calculating or comparing measures of diagnostic accuracy” [88]; this systematic review aimed to focus on description of methods for MRMC studies using ROC AUC as an outcome measure.

The large majority of studies used less than 10 observers, some did not describe reader experience, and the majority did not mention whether observers were aware of prevalence of abnormality, a factor that may influence diagnostic vigilance. Most studies required readers to detect lesions while a minority asked for characterization, and others were a combination of the two. We believe it is important for readers to understand the precise nature of the interpretative task since this will influence the rating scale used to build the ROC curve. A variety of units of analysis were adopted, with just under half being the patient case. We were surprised that some studies failed to record the number of disease-positive and disease-negative patients in their dataset. Concerning the confidence scales used to construct the ROC curve, only a small minority (12%) of studies stated that readers were trained to use these in advance of scoring. We believe such training is important so that readers can appreciate exactly how the interpretative task relates to the scale; there is evidence that radiologists score in different ways when asked to perform the same scoring task because of differences in how they interpret the task [89]. For example, readers should appreciate how the scale reflects lesion detection and/or characterization, especially if both are required, and how multiple abnormalities per unit of analysis are handled. Encouragement to use the full range of the scale is required for normal rating distributions. Whether readers must use the same scale for patients with and without pathology is also important to know.

Despite their importance for understanding the validity of study results, we found that description of the confidence scores, the ROC curve and its analysis was often incomplete. Strikingly, only three studies described the distribution of confidence scores and none stated whether transformation to a normal distribution was needed. When publically available DBM MRMC software (ref DBM) is used for ROC AUC modeling, this requires assumptions of normality for confidence scores or their transformations when ROC curve fitting methods are used. Where confidence scores are not normally distributed these software methods are not recommended [84]–[86], [90]. Although Hanley shows that ROC curves can be reasonable under some distributions of non normal data [91], concerns have been raised particularly in imaging detection studies measuring clinically useful tests with good performance to distinguish well defined abnormalities. In tests with good performance two factors make estimation of ROC AUC unreliable. Firstly readers' scores are by definition often at the ends of the confidence scale so that the confidence score distributions for normal and abnormal cases have very little overlap [82]–[86]. Secondly tests with good performance also have few false positives making ROC AUC estimation highly dependent on confidence scores assigned to possibly fewer than 5% or 10% of cases in the study [86].

Most studies did not describe the method used for curve fitting. Over 40% of studies presented no ROC curve in the published article. When present, the large majority were smoothed and averaged over all readers. Only four articles presented data points underlying the curve meaning that the degree of any extrapolation could not be assessed despite this being an important factor regarding interpretation of results [92]. While, by definition, all studies used MRMC AUC methods, most reported additional non-AUC outcomes. Approximately one-quarter of studies did not present AUC data for individual readers. Because of this, variability between readers and/or the effect of individual readers on the ultimate statistical analysis could not be assessed.

Interpretation of study results was variable. Notably, when no significant change in AUC was demonstrated, authors stated that the number of cases was either insufficient or that the difference could not be resolved by the study, appearing to claim that their studies were underpowered rather than that the intervention was ineffective when required to improve diagnostic accuracy. Indeed some studies claimed an advantage for a new test in the face of a non-significant increase in AUC, or turned to other outcomes as proof of benefit. Some interpreted no significant difference in AUC as implying equivalence.

Our review does have limitations. Indexing of the statistical methods used to analyse studies is not common so we used a proxy to identify studies; their citation of “key” references related to MRMC ROC methodology. While it is possible we missed some studies, our aim was not to identify all studies using such analyses. Rather, we aimed to gather a representative sample that would provide a generalizable picture of how such studies are reported. It is also possible that by their citation of methodological papers (and on occasion including a ROC researcher as an author), our review was biased towards papers likely to be of higher methodological quality than average. This systematic review was cross-disciplinary and two radiological researchers performed the bulk of the extraction rather than statisticians. This proved challenging since the depth of statistical knowledge required was demanding, especially when details of the analysis was being considered. We anticipated this and piloted extraction on a sample of five papers to determine if the process was feasible, deciding that it was. Advice from experienced statisticians was also available when uncertainty arose.

In summary, via systematic review we found that MRMC studies using ROC AUC as the primary outcome measure often omit important information from both the study design and analysis, and presentation of results is frequently not comprehensive. Authors using MRMC ROC analyses should be encouraged to provide a full description of their methods and results so as to increase interpretability.

## Supporting Information

S1 File **Extraction sheet used for the systematic review.**
(DOC)

S2 File **Raw data extracted for the systematic review.**
(XLS)

S1 PRISMA Checklist (DOC)
