# Supplementary material for: Multi-Reader Multi-Case Studies Using the Area under the Receiver Operator Characteristic Curve as a Measure of Diagnostic Accuracy: Systematic Review with a Focus on Quality of Data Reporting
Source: PLoS One. 2014 Dec 26;9(12):e116018. doi: 10.1371/journal.pone.0116018 (PMC4277459; doi:10.1371/journal.pone.0116018)
Supplement: S1 File — Extraction sheet used for the systematic review. (DOC) [file pone.0116018.s001.doc]

**S1:**

**Data extraction form used for MRMC ROC AUC systematic review**

Feb 2013

♣ indicates hidden text entry explaining data extraction sheet. To view use tools, options and tick hidden text box.

**A.** Review details

| Reviewer: |  |
| --- | --- |
| Date of data extraction: |  |
| Paper number: |  |
| Author of paper: |  |
| Year of publication: |  |
| Journal: |  |
| Vol and page no: |  |

**B.** Inclusion criteria

| **Does the paper fulfil the following criteria:** | **Yes** | **No** | **Page No** |
| --- | --- | --- | --- |
| B1 Cites Dorfman 1992 paper or other methodological papers related to (see below) |  |  |  |
| B2 Reports attempting to use an MRMRC ROC (AUC) method (DBM one) |  |  |  |
| B3 Published 2005 onwards |  |  |  |
| B4 Uses human readers (not test of machine alone) |  |  |  |
| B5 Uses real patient data, not simulated lesions |  |  |  |
| B6 Diagnostic test accuracy study |  |  |  |
| B7 Primary study with original data (not review, methodology development etc) |  |  |  |
| B8 Using these ROC AUC methods is a main aims of the paper |  |  |  |

♣ Dorfman DD, Berbaum KS, Metz CE. Receiver operating characteristic rating analysis. Generalization to the population of readers and patients with the jackknife method. Invest Radiol. 1992 Sep;27(9):723-31.

Dorfman DD, Berbaum KS, Lenth RV, Chen YF, Donaghy BA. Monte Carlo validation of a multireader method for receiver operating characteristic discrete rating data: factorial experimental design. Acad Radiol. 1998 Sep;5(9):591-602.

Hillis SL, Berbaum KS. Monte Carlo validation of the Dorfman-Berbaum-Metz method using normalized pseudovalues and less data-based model simplification. Acad Radiol. 2005 Dec;12(12):1534-41.

Hillis SL, Berbaum KS. Power estimation for the Dorfman-Berbaum-Metz method. Acad Radiol. 2004 Nov;11(11):1260-73.

Hillis SL, Obuchowski NA, Schartz KM, Berbaum KS. A comparison of the Dorfman-Berbaum-Metz and Obuchowski-Rockette methods for receiver operating characteristic (ROC) data. Stat Med. 2005 May 30;24(10):1579-607.

Hillis SL. A comparison of denominator degrees of freedom methods for multiple observer ROC analysis. Stat Med. 2007 Feb 10;26(3):596-619.

Metz CE. ROCKIT. Chicago, Ill: Department of Radiology, University of Chicago, 2007.

Obuchowski NA, Rockette HE. Hypothesis testing of diagnostic accuracy for multiple readers and multiple tests: an ANOVA approach with dependent observations. Comm Stat Sim Comput 1995;24:285-308

Obuchowski NA. Multireader multi-modality ROC studies: hypothesis testing and sample size estimation using an ANOVA approach with dependent observations with rejoinder. Acad Radiol 1995; 2:S22-S29

B2 Seen in the methods section as a clear attempt to provide a number measuring accuracy.

.♣

**C. Data extraction**

| **C. Characteristics of studies included in review** | **Code** | **Page number** |
| --- | --- | --- |
| C1 Disease (e.g. breast cancer, breast calcification) |  |  |
| C2 Nature of diagnosis [1=characterisation of polyp e.g. malignant vs benign, 2=presence/absence, 3=both 1 and 2, U, NR] |  |  |
| Tests |  |  |
| C3 Index test method [0=Unclear, 1=imaging, 3=other]  If other specify test |  |  |
| C4 How many tests or methods does article compare to reference standard? |  |  |
| C5 Reference method [0=Unclear, 1=imaging, 2=histological findings, 3=clinical and/or biological and/or radiological follow up, 4=mixture (specify), 5=other (specify)] |  |  |
| Patients |  |  |
| C6 Clinical population of patients:  C6a Study design used [0=Prospective study designed for that purpose, 1=Secondary analysis of a previous prospective study, 2=retrospective study, 3=Random selection from clinical pop, 4= Other, U, NR]. Specify if other.  C6b Single/Multicentre [0=Single, 1=Multi, U, NR]  C6c Are patients symptomatic or asymptomatic? [A=asymptomatic, S=symptomatic, U, NR] |  |  |
| C7 Is the source of patients reported (geographical region or name of hospital)? [Y, N, U] |  |  |
| C8a After inclusion criteria, is there any apparent selection other than by type of disease/ time/ place (no selection=consecutive recruitment given inclusion criteria)? [1=apparently unselected, 2=selected & give some criteria, 3= U]  If selection then  C8b. Which type of selection is applied? [0=no apparent selection, 1=to reproduce clinical daily frequency of disease among a screened population, 2=to enrich prevalence but to retain disease severity spectrum (e.g. randomly from cases and diseases), 3=to select subtle and difficult cases to interpret, 4=other, 5= U/NR]  C8c Describe how selection is made: based on experts’ opinion regarding the subtlety/difficultness of the reading? [NA, text] |  |  |
| C9 Description of patients reported (minimum age, and gender for Y) [Y, N, U] |  |  |
| Readers |  |  |
| C10 Number of readers [NR] |  |  |
| C11 Is there sufficient reporting of prior experience of readers? (i.e. minimum number of individual cases or number of years in practice reported for inclusion or as description of readers) [Y, N, U] |  |  |
| C12 During the study, is a specific training of readers reported? [Y, N, U, NR] |  |  |
| Avoidance of bias in study design |  |  |
| C13 Were the readers blinded to patients’ characteristics and lesion prevalence in the sample (if the tested hypothesis was not the impact on accuracy on such information)? [Y blinded to both, Y blinded to prevalence, Y blinded to clinical info., N, U, NR] |  |  |
| C14 Do readers read the same patient more than once (e.g. with different index tests) ? [Y, N, U,NR]  If Y then...  C14a Was there a time interval between the different readings (to prevent recall bias)? [Y, N, U, NR, NA if readers do not read same case more than once, NA if readers do not read same case more than once]  C14b When the same patient is read more than once using different index tests, was the order of patients the same? [,Y, N U, NR]  C14c Was the order of different index tests the same for each patient? [Y, N, U, NR]  C14d Was a fully crossed design used? (all readers read all images in all modalities/index tests) [Y written, Y obvious but not written, N written, N obvious but not written, U, NR] |  |  |
| Units of analysis and sample size |  |  |
| C15a Unit of analysis for ROC AUC [0=NR/U, 1=patients, 2=segments, 3=organ, 4=lesion, 5=other]  C15b Unit of analysis for other outcomes [0=NR/U, 1=patients, 2=segments, 3=organ, 4=lesion, 5=other]  C15c What is the nature of unit of analysis? [1=single image, 2=video, 3=multiple images, 5=multiple modalities (e.g. CT plus MRI), 6=NR/U |  |  |
| C16 Sample size: DN  C16a Number DN patients  C16b Number DN in units for first outcome in paper [Delete as applicable: examinations/ organs /lesions/ other] |  |  |
| C17 Sample size: DP  C17a Number DP patients  C17b Number DP in units for first outcome in paper  [Delete as applicable: examinations/ organs /lesions/ other] |  |  |
|  |  |  |
|  |  |  |
| C20 Comments | | |

♣ U, Unclear; NR, not reported.

C2 Screening if the patients are at risk of disease but not symptomatic; diagnosis if the patients of the setting are symptomatic and need that test to diagnose or rule out the specific disease.

C5 Trying to find out which reference tests are based on pathology/follow up/other imaging vs expert opinion but where test is to invasive to be used to give definitive result. An example is lung cancer where a solitary nodule is judged to be non cancerous based on expert opinion as biopsy is too invasive. Contrast to colon cancer, where colonoscopy is done on all patients as a reference standard, and reference standard is not an expert consensus of the CT colonography alone. Note there are uncertainties of ref standard and in many cases incorporation bias issues where there is not definitive alternative test for reference standard. Restricted to the cases.

Yes if the reference test is the same examination read by several experts and based on their opinion, answer not if something different (even if read by expert).

C8 Were any additional inclusion criteria used e.g. all easy cases, difficult cases, cases where there has been previous disagreement between radiologists. Interested in selection bias that may have affected the diagnostic accuracy of the test that the authors looked at.

C15aUnit of analysis - what is the basis for the scoring - one score per film, one per lesion, one per body segment, one per region of interest?♣

| **D. Methods of recording study outcomes** | **Code** | **Page number** |
| --- | --- | --- |
| D1 Are particular lesions/ ROI presented to readers for scoring compared to set of images as used in normal clinical practice? [Y, N, NR, U] |  |  |
| D2a What is nature of confidence score? [0=NR/U, 1= benign/malignant (or other nature of a lesion), 2=presence/absence of lesion (whatever its nature), 3= malignant/benign/no lesion, 4=other]  D2b If another score is given to assess test accuracy (e.g.BIRADS), what is nature of that score? [0=NR/U, 1= benign/malignant (or other nature of a lesion), 2=presence/absence of lesion (whatever its nature), 3= malignant/benign/no lesion, 4=other]  D2c Do all units examined have a lesion/nodule (e.g. if units of analysis per patient, then do all patients have a lesion)? [Y, N, NR, U] |  |  |
| D3a What is basis of confidence scores used in ROC AUC calculation? [0=NR, 1=1 to 5/10 categories, 2=continuous percentage, 3=continuous percentage but with categories, 4=other, 5=U]  If categories scale, extract the number of categories in the scale.  D3b Are readers instructed/trained as part of study to use confidence score? [Y, N, NR, U]  D3c Is there a method to encourage “full use of confidence score” as recommended by FDA? [Y, N, NR, U] |  |  |
| D4a Are two types of TN reported? [Y, N, U]  D4b Are there two types of TN? [Y, N, U] |  |  |
| D5 If two types of TN reported, are they scored differently? [Y, N, U, NR] |  |  |
| D6a Are there multiple lesions/polyps etc in unit of analysis tested? [Y, N, U, NR]. State if it is written (W) or obvious but not written (O) if Y or N.  D6b If so, how are these multiple lesions treated? Comment. |  |  |
| D7 Comments | | |

♣ D1Were particular ROIs (region of interest)/lesions identified to readers and they were just ranking those on a confidence scale (classification task only) or were readers looking at an image, identifying if there were any lesions and then classifying them.

D3b All variations for a high confidence report could be (Harrington, 1990):

- the image depicts the finding clearly (ie, the image quality is good)
- the finding is a pathology that is clearly apparent whatever the quality of the image (ie, the finding is obvious)
- the finding is common among patients of this sex, history, and presenting complaint (ie, the finding is both suggested and supported by extra-image information)
- the finding could be one of two or more things, one far more common in patients like the present one (ie, the finding’s existence is judged against its estimated likelihood of occurrence)
- the finding is serious, if actually present, and should be verified in other way (ie, the existence of the finding is judged against its potential risk to the patient)
- the finding is a pathology seen many times and, therefore, is readily recognized even in images of mediocre quality (ie, the finding is familiar)
- the finding is one which, if not reported, might be a factor in a subsequent malpractice suit (ie, the finding is good defensive medicine)
- though not especially well depicted, the finding id usually present when a related, well-depicted finding is evident (ie, the finding is part of a common syndrome in patients like the present one)
- the pathology is apparent on a familiar viewing medium though it might not be on a new medium (ie, the finding is apparent, in part, because the viewing medium is familiar)
- the finding might be missed by others, but I can spot the tough ones (ie, the confidence of the radiologist concerning his or her professional skills is high)

D4 Types of TN defined as 1/ TN that were not dwelled by readers, 2/ those that were dwelled but been decided as negative.

D5 Are these TN scored differently from FP, FN and TP? ♣

| **E. Model assumptions** | **Code** | **Page number** |
| --- | --- | --- |
| E1 Is there one type of confidence score all assigned by reader? [0=NR/U, 1=one score for all cases, 2=one score for positive reported cases and no mention of how the rest was rated, 3=one score for positive reported cases and one other score for negative reported cases,4=other] |  |  |
| E2a Are confidence scores transformed to make normally distributed? [Y, N, U, NR]  E2b Is the distribution of confidence scores reported? [Y, N, U, NR]  E2c If reported, is the distribution normal? [Y, N, U, NR] |  |  |
| E3 Is it reported that the data fulfilled the assumptions of the modelling (i.e. were normal)? [Y, N, U, NR] |  |  |
| E3b Comment | | |
| **E. Model fitting** | **Code** | **Page number** |
| E4a Are ROC curves presented? [Y, N, U]  E4b If so, which ROC curves are presented? [1=for each reader, 2=average over readers, 3=other, NC=no curve, U, NR]  E4c If ROC curves presented, are data points underlying the ROC shown? [Y, N, U]  E4d Is the ROC curve smooth (i.e. fitted)? [Y, N, U]  E4e Did AUC calculation use unreasonable extrapolation beyond data (i.e. no data points in last quarter on RHS of ROC plot)? [Y, N, U] |  |  |
| E5 Is partial AUC used as an outcome? [Y, N, U] |  |  |
| E6 What method is used for curve fitting? [0=NR/U, 1=parametric (ML), 2=non parametric (Trapezoidal/Wilconxon), 3=Proproc method, 4=other]. If other, specify. (See note on software) |  |  |
| E7 Were ROC curves fitted and/or AUC calculated for each reader? [Y, N, U]  E7a Were any problems reported with curve fitting? [Y, N, U]  E7b Is one or more data set said to be degenerate (methods, results, discussion)? [Y, N, NR]. If so, cut and paste relevant sentences. |  |  |
| E8 What method is used to compare AUC outcomes? [0=NR/U, 1=complex AUC MRMC methods 2=t-test of AUC, 3=other].  If other, specify. |  |  |
| E9 What method is used to compare **non AUC** outcomes? [0=NR/U, 1=multilevel methods, 2=t-test of outcomes, 3=other].  If not t-test then specify. |  |  |
| E10a Did the study include an author from methodological group that developed method e.g. Metz lab [Y, N, U] (see list below).  E10b Or in the acknowledgements section? |  |  |
| E11 What key methodological authors were cited? |  |  |
| E12 Comments | | |

♣ E1 - Answer from our assessment of the paper, as this will only rarely be reported. Basically could a lesion be seen but it is e.g. benign (one type of TN) but also could be that no lesion is seen in image that can be categorised as benign/malignant (other type of TN). E1 question can be answered by mentioned 3 situations:

- Readers were instructed to provide a confidence rating to the cases, and then gave a score to all cases (1)

- Readers were instructed to provide a confidence rating if they saw something. Then they gave a score to all the cases they considered as positive ones, and nothing to the others, which were attributed (according to the statistical analysis paragraph) a 0 for example (2)

- Readers were instructed to provide one confidence rating to the “lesion” they saw/considered as a lesion, and another confidence rating when they saw nothing (to score their confidence in seeing nothing) (3).

E2a We are expecting a graph, or mean and SD, or median with range or IQR.

E4 If Assumptions of the model metioned somewhere:

- are they mentioned in the methods section?
- Is the assumption regarding the normality of distributions of confidence scores mentioned in the methods section?
- Is this assumption reported as fulfllied by the data in the results section?

E7 Rscore, Rockit, LabROC5: to fit ROC curves with conventional method (i.e. bi-normal model)

Proproc: to fit ROC curves using proper binormal model

CORROC, CLABROC, ROCKIT: to fit ROC curves (continuous or not data) then compare modalities, but no generalisation across readers

LabMRMC, DBM-MRMC: to fit ROC curves, then compare modalities in MRMC.

LabMRMC: use either parametric model (ML model), either non-parametric method (empirical, Wilcoxon, Trapezoidal). Deals with degeneracy if small and sparse by replacing fitted AUC by empirical values.

MRMC 2.1 B2 uses either parametric models (ML model), either non-parametric method (trapezoidal/Wilcoxon), either non conventional binormal models such as Proper method (incorporated into the software, no need to use separately Proproc software) or CBM method.

E8

E10 – Author from:

- Methodological Metz lab group: CE Metz; DD Dorfman; University of Chicago.
- HE Rockette, Department of Biostatistics, Graduate School of Public Health, University of Pittsburgh, Pittsburgh, PA.
- NA Obuchowski, H Ishwaran, HH Song, Departments of Bioistaticitcs and Epidemiology, and Radiology, Cleveland Clinic Foundation, Cleveland, OH.
- S Hillis, KS Berbaum, University of Iowa, IO.
- SV Beiden, , RF Wagner, Center for Devices & Radiological Health, Food and Drug Administration, Rockville,Maryland
- AY Toledano, Center for Statistical Sciences, Department of Community Health, Brown University, Providence, Rhode Island .
- K Doi, Kurt Rossman laboratories for Radiologic Images, Image Research, Department of Radiology, University of Chicago, Chicago. ♣

| **F. How are results presented?** | **Code** | **Page number** |
| --- | --- | --- |
| F1 Which accuracy measures are reported in methods/results section? [0= none, 1=AUC, 2=∆AUC, 3=sens/spec, 4=PPV/NPV, 5=LR+/ LR-] |  |  |
| F2a Is ∆AUC used as primary outcome? [Y, N, U, NR]  F2b If not, what is the primary outcome measure [0=sens/spec, 1=other]. If other specify. |  |  |
| F3 Are individual reader results reported (e.g AUC, ∆AUC)? [Y, N, U] |  |  |
| F4 Are absolute average outcome measures reported [0=NR/U, 1=AUC and/or sens/spec for both tests, 2= difference in outcome between tests only, 3= one outcome difference only, other each test result, 4=other] |  |  |
| F5 What is the size of the ∆AUC averaged across readers? |  |  |
| F6 Is a significant ∆AUC reported in study? [Y (significant result), N (non significant result), U, NR] |  |  |
| F7 Is this significant ∆AUC result likely to be due to the results in a single reader or patient? [Y, N, U, Not possible to say, NA] |  |  |
| F8 If ∆AUC is increased but not significant, is it reported as showing an advantage of a particular test? [Y, N, NR, U, NA] |  |  |
| F9 Comments | | |

♣

F1 Use as many options as needed e.g. if AUC and either sens or spec are used, then report 1, 3. Use option 2 even if just a p-value is given for ∆AUC or if a comment is made that the AUC from two methods is the same.

Primary outcome is defined as the analysis that was mentioned in the article as the primary outcome. Alternatively if there is no mention of which analysis is the primary outcome this is the analysis that appeared to be reported the first one in the article: in the title, or then in the abstract, or then in the study aim, or then in the results section.

F2aChange in AUC (∆AUC) is taken as primary outcome if specifically mentioned or if it is the first outcome reported in title or if not in title then in abstract, or if no outcomes reported in either title or abstract, then is it the first outcome reported in the results

F3 Do they give the results for individual readers or only results averaged over all readers?

F4 Want to know which articles give actual values for AUC (or sens/spec) and which give only the difference in AUC between two tests.

F11b When ROC curve is not smooth, then it is jagged line.

♣

| G. Conclusions/ recommendations? | **Code** | **Page number** |
| --- | --- | --- |
| G1 Is ∆AUC interpreted clinically in terms of patient diagnosis (eg TP, FP, etc)? [Y, N, NR, U] |  |  |
| G2 Is ∆AUC a non significant difference? [Y, N, U]  If Y, then  G2a Do the authors give a reason why ∆AUC has no change? [Y, N, U, NA]  G2b Do authors do the following [1=make main discussion a significant change in sens and or spec, 2=turn conclusion into an interpretation of equivalence of two tests, 3= switch to a general comment on the accuracy of the test method, ignoring ROC AUC results, 4=other, please specify, 5=NA] |  |  |
| G3 Comments | | |

♣ G1 Is there a translation of the results in clinically comprehensive wording for patients and physicians? More explicated ad detailed than “this test method had a good accuracy”. ♣

**General notes**

If more than one comparison of method tests (e.g. 2 by 2 comparison), please extract all comparison, using several spreadsheet if necessary

If more than one scale used in the paper for the confidence rating, please extract data regarding the one on which the ROC analysis is based. If several scales are used for that purpose, please extract all data using several spreadsheet if necessary.

If more than one disease picked up (e.g. masses in one hand and clusters of micro-calcifications in another one), please extract all the data using several spreadsheet if necessary.

**Comments**
